# Evaluation of left atrial function in patients with iron-deficiency anemia by two-dimensional speckle tracking echocardiography

**DOI:** 10.1186/s12947-016-0078-z

**Published:** 2016-08-23

**Authors:** Jiaqi Shen, Qiao Zhou, Yue Liu, Runlan Luo, Bijun Tan, Guangsen Li

**Affiliations:** Department of Ultrasound, The Second Affiliated Hospital of Dalian Medical University, Dalian, 116027 China

**Keywords:** Two-dimensional speckle tracking echocardiography, Left atrial function, Iron-deficiency anemia

## Abstract

**Background:**

Iron-deficiency anemia (IDA) is a global health problem and a common medical condition that can be seen in everyday clinical practice. And two-dimensional speckle tracking echocardiography (2D-STE) has been reported very useful in evaluating left atrial (LA) function, as well as left ventricular (LV) function. The aim of our study is to evaluate the LA function in patients with IDA by 2D-STE.

**Methods:**

65 patients with IDA were selected. This group of patients was then divided into two groups according to the degree of hemoglobin: group B (Hb > 90 g/L) and group C (Hb60 ~ 90 g/L). Another 30 healthy people were also selected as control group A. Conventional echocardiography parameters, such as left atrial diameter (LAD), peak E and A of mitralis (E, A), E/A, end-diastolic thickness of ventricular septum (IVST d), end-diastolic thickness of LV posterior wall (PWTd) and left ventricular end-diastolic dimension (LVDd) were obtained from these three groups. Left atrial minimum volume (LAVmin), left atrial pre-atrial contraction volume (LAVp) and left atrial maximum volume (LAVmax) were measured by Simpson’s rule, whereas left atrial active ejection fraction (LAAEF) and left atrial passive ejection fraction (LAPEF) were obtained from calculation. Two-dimensional images were acquired from apical four-chamber view and two-chamber view to store images for offline analysis. The global peak atrial longitudinal strain and strain rate of systolic LV (GLSs, GLSRs) as well as early and late diastolic LV strain rate (GLSRe, GLSRa) curves of LA were acquired in each LA segment from basal segment to top segment of LA by 2D-STE.

**Results:**

Compared with group A, there were no differences between group B and group A (all *P* > 0.05). The LAAEF and GLSRa were significantly higher in group C compared with those of group A and group B (all *P* < 0.01). The LAPEF, GLSs, GLSRs and GLSRe were significantly lower in group C compared with those of group A and group B (all *P* < 0.01).

**Conclusions:**

2D-STE could evaluate the LA function in patients with IDA.

## Background

World Health Organization (WHO) defines Anemia as hemoglobin levels < 13 g/dL (hematocrit < 39 %) in males, < 12 g/dL (hematocrit < 36 %) in non-pregnant females, and < 11 g/dL (hematocrit <33 %) in pregnant females [1]. Iron deficiency and iron-deficiency anemia (IDA) are global health problems and common medical conditions that can be seen in everyday clinical practice [2]. WHO estimates that 42 % of pregnant women, 30 % of non-pregnant women (aged 15 to 50 years), 47 % of preschool children (aged 0 to 5 years), and 12.7 % of men older than 15 years worldwide are anemic [3]. Anemia affects one-fourth of world’s population, accounted for 8.8 % of the total global burden of disease [4]. Iron deficiency is the predominant cause of anemia across countries, with women more commonly afflicted than men [4]. Although the prevalence of iron-deficiency anemia somehow has recently declined, iron deficiency continues to be the top-ranking cause of anemia worldwide. Iron deficiency may result from inadequate iron intake and absorption, increased iron requirements for growth, and excessive iron losses [5].

Based on the physiological significance of oxygen transported to myocardial tissue, anemia may be a cause of more severe cardiovascular diseases or a sign of other severe diseases that occur in the body. The physiologic response to anemia is a compensatory increase in cardiac output in order to maintain adequate oxygen delivery [6]. Patients are asymptomatic with mild anemia. Dyspnea and fatigue may occur when anemia is further aggravated. In severe cases, iron-deficiency anemia can lead to LV dysfunction and heart failure [6]. It has reported that myocardial contractility would decrease when hemoglobin was below 7 g/dL [7] and chronic anemia would result in increased LV end-diastolic pressure as well as decreased functional reserve [8]. Frequent research has been conducted on LA structural and functional remodeling, which is a cause of LV diastolic dysfunction, therefore the significance of LA has drawn much attention nowadays [9]. Increased left atrial size has been shown as an important predictor of target organ damage and multiple adverse cardiovascular events [10, 11].

2D-STE is a new technology to accurately evaluate LA function in normal subjects [12]. It has the advantage in accurate quantification of myocardial deformation and being angle independent [13, 14]. Hence the goal of the research is to evaluate the LA function in patients with IDA by 2D-STE.

## Methods

### Study population

Between November 2014 and April 2016, we studied 65 patients between the age of 22 and 65 with IDA, (male: female = 1:5.5). 38 patients with IDA were caused by gynecological disease such as uterine leiomyoma, adenomyosis and increased menstrual flow. 18 patients with IDA were caused by digestive system disease such as subtotal gastrectomy. Another 9 cases with IDA were caused by unknown reasons. We rejected other causes of heart disease such as coronary heart disease, hypertension, congenital heart disease, diabetes mellitus, systemic lupus erythematosus, cardiopulmonary surgery and any grade of valvular stenosis etc. According to the degree of hemoglobin, 65 patients were classified into mild group (34 patients, 90 g/L ≤ Hb < 120 g/L, aged 25–65 years, mean age: 48.8 ± 14.1 years, male: female = 7:27, IDA duration were all between 7 months and 15 years,mean time: 6.5 ± 3.3 years) and moderate group (31 patients, 60 g/L ≤ Hb < 90 g/L aged 22–62 years, mean age: 47.6 ± 16.6 years, male: female = 6:25, IDA duration were all between 8 months and 14 years,mean time: 6.2 ± 3.6 years). In addition, we did not have enough patents with severe IDA as patients with hemoglobin below 60 g/L were very rare to find, and in most of all cases were being cured immediately, thus their duration at severe anemia stage did not last long. The control group consisted of 30 healthy volunteers (aged 23–64 years, mean age: 47.0 ± 14.5 years, male: female = 6:24). All of them had no cardiovascular diseases with all examinations results shown as normal. This study was consented by the Second Affiliated Hospital’s Ethics Committee of Dalian Medical University on human research and all patients were being informed and consented to participate in this research.

### Clinical and laboratory examination

All patients had completed a physical examination, From which height and weight were measured, and body mass index (BMI, kg/m^2^) and body surface area (BSA, m^2^) were calculated. Blood pressure and blood glucose were measured before echocardiographic examination. A 12-lead standard resting electrocardiogram (ECG) was performed among all patients.

### Conventional echocardiography

An ultrasound system (Vivid E9, GE Medical Health, USA) and an M5S-D probe (1.5–4.5 MHz) were used for the study. Every subject had a conventional echocardiography examination in sinus rhythm. Every subject was connected with ECG and was in the left lateral decubitus position, eupnea. The parameters were measured by conventional echocardiography, such as left ventricular end-diastolic dimension (LVDd), end-diastolic thickness of ventricular septum (IVSTd), end-diastolic thickness of LV posterior wall (PWTd) and left atrial chamber dimension (LAD). Early (E) and late (A) diastolic mitral inflow velocities were measured by pulsed wave Doppler, and E/A ratio was also calculated. Maximal, preatrial contraction, and minimal LA volume were acquired by the biplane modified Simpson’s rule [15]. The maximum LA volume (LAVmax) was obtained before standard mitral valve opening. The precontraction LA volume (LAVp) was obtained at the precise beginning of the ECG P wave, and the minimum LA volume (LAVmin) was obtained precisely at the late diastolic left ventriculus. Then left atrial active ejection fraction (LAAEF) and left atrial passive ejection fraction (LAPEF) were calculated using the following formulas [16, 17]: passive emptying index (LAPEF) was calculated by ([LAVmax-LAVpre-a]/LAVmax), active emptying index (LAAEF) was calculated by ([LAVpre-a-LAVmin]/LAVpre-a). Standard apical four- and two-chamber views were acquired according to the guidelines of the American Society of Echocardiography (3 consecutive heart cycles). Images were stored for offline analysis.

### Speckle tracking

Standard images obtained in 2D mode were analyzed using the EchoPAC software. Frame rates were controlled between 40 and 60 frames/sec. The endocardial interfaces of left atrial were demonstrated completely and they were traced manually by using a point-and-click method at the end of atrial contraction. Epicardial surface tracing were then generated automatically by the software and were changed manually based on the thickness of LA wall. The LA wall was then divided into 6 segments automatically. If some segments were unavailable due to unsatisfactory tracking quality, these images would be removed. At the end, the final results were acquired in both four and two chamber views. Longitudinal strain and strain rate curves were generated for global LA wall. Global peak LA longitudinal strain rate of early and late diastolic LV (GLSRe, GLSRa), as well as the global peak LA longitudinal strain and strain rate of systolic LV (GLSs, GLSRs) were obtained. Last but not the least, the peak longitudinal systolic strain of LV was obtained and their absolute values were compared.

### Statistical analysis

The data were analyzed with SPSS 17.0 for Windows system. Numeric variables were presented as mean ± standard deviation (SD). One-way Analysis of Variance (ANOVA) was performed to test for statistically significant differences among the four groups. Continuous data were compared between differences among individual groups using the Student-Newman-Keuls post-test. All statistical tests were two sided, and *p* < 0.01 was set for statistical significance. Intra-observer analysis of global longitudinal strain and strain rate in the 4-chamber view were conducted two months after completion of the initial measurements (SJQ). For inter-observer variability, a second observer (LGS) analyzed 20 % of the initial images. Intra-observer variability and inter-observer variability were assessed using the intra-class correlation coefficient (ICC).

## Results

### Demographic and clinical characteristics

Demographic and clinical characteristics of the three groups are presented in Table 1. There were no significant differences among the three groups with respect to age, gender, duration of IDA, heart rates, body mass index, systolic arterial pressure, diastolic arterial pressure or blood glucose level (all P > 0.05).

**Table 1:** Demographic and clinical characteristics of the study population

Demographic characteristics/Risk factors	Group A	Group B	Group C
	(*n* = 30)	(*n* = 34)	(*n* = 31)
Male: female ratio	6:24	7:27	6:25
Age, years (mean ± SD)	47.0 ± 14.5	48.8 ± 14.1	47.6 ± 16.6
Duration of IDA (years)	0	6.5 ± 3.3	6.2 ± 3.6
Heart rate(rates/min)	71 ± 12	72 ± 9	70 ± 10
Body mass index(kg/m2)	22.4 ± 2.6	23.2 ± 2.9	22.5 ± 2.7
Systolic arterial pressure(mmHg)	120 ± 11	123 ± 13	121 ± 14
Diastolic arterial pressure(mmHg)	73 ± 7	75 ± 9	73 ± 10
Blood glucose (mmol/L)	4.52 ± 0.37	4.73 ± 0.42	4.62 ± 0.28

### Traditional echocardiographic parameters

There were no significant differences between group B and group A (all *P* > 0.05). None of LVDd, IVSTd and PWTd were significant among the three groups. The LAD, LAVp, LAVmax, LAVmin, A and LAAEF of group C were significantly higher than those of groups A and B. E, E/A and LAPEF of group C were significantly lower than those of groups A and B (all *P* < 0.01) (Seen in Table 2).

**Table 2:** Echocardiographic characteristics of the study populations

Variables	Group A	Group B	Group C
	(*n* = 30)	(*n* = 34)	(*n* = 31)
LAD(mm)	32.69 ± 2.43	34.00 ± 2.80	37.49 ± 1.61*#
LVDd(mm)	43.51 ± 2.45	43.90 ± 2.36	44.35 ± 2.92
IVSTd(mm)	8.79 ± 0.67	9.10 ± 0.41	9.20 ± 0.60
PWTd(mm)	8.82 ± 0.76	9.06 ± 0.33	9.16 ± 0.53
E velocity(m/s)	0.96 ± 0.15	0.90 ± 0.18	0.72 ± 0.12*#
A velocity(m/s)	0.77 ± 0.15	0.78 ± 0.12	1.05 ± 0.16*#
E/A	1.13 ± 0.39	1.14 ± 0.34	0.70 ± 0.06*#
LAVmax(ml)	35.53 ± 2.66	37.09 ± 3.51	55.71 ± 8.72*#
LAVmin(ml)	14.19 ± 1.32	14.47 ± 1.39	22.27 ± 3.93*#
LAVp (ml)	20.66 ± 1.54	20.65 ± 1.50	36.92 ± 5.57*#
LAPEF(%)	41.87 ± 3.23	43.33 ± 2.58	33.33 ± 2.60*#
LAAEF(%)	31.42 ± 2.39	29.95 ± 3.18	40.01 ± 6.17*#

*LAD* left atrial dimension, *LVDd* left ventricular end-diastolic diameter, *IVSTd* end-diastolic thickness of ventricular septum, *PWTd* end-diastolic thickness of LV posterior wall, *E/A* ratio of peak early and

late diastolic velocities, *LAVmax* Left atrial maximum volume, *LAVmin* LA minimum volume, *LAVp* LA pre-atrial volume, *LAPEF* left atrial passive emptying fraction, *LAAEF* left atrial active emptying fraction. *P <0.01 versus the control group. #*P* <0.01 versus the mild group

### Left atrial strain and strain rate

**Fig. 1:**
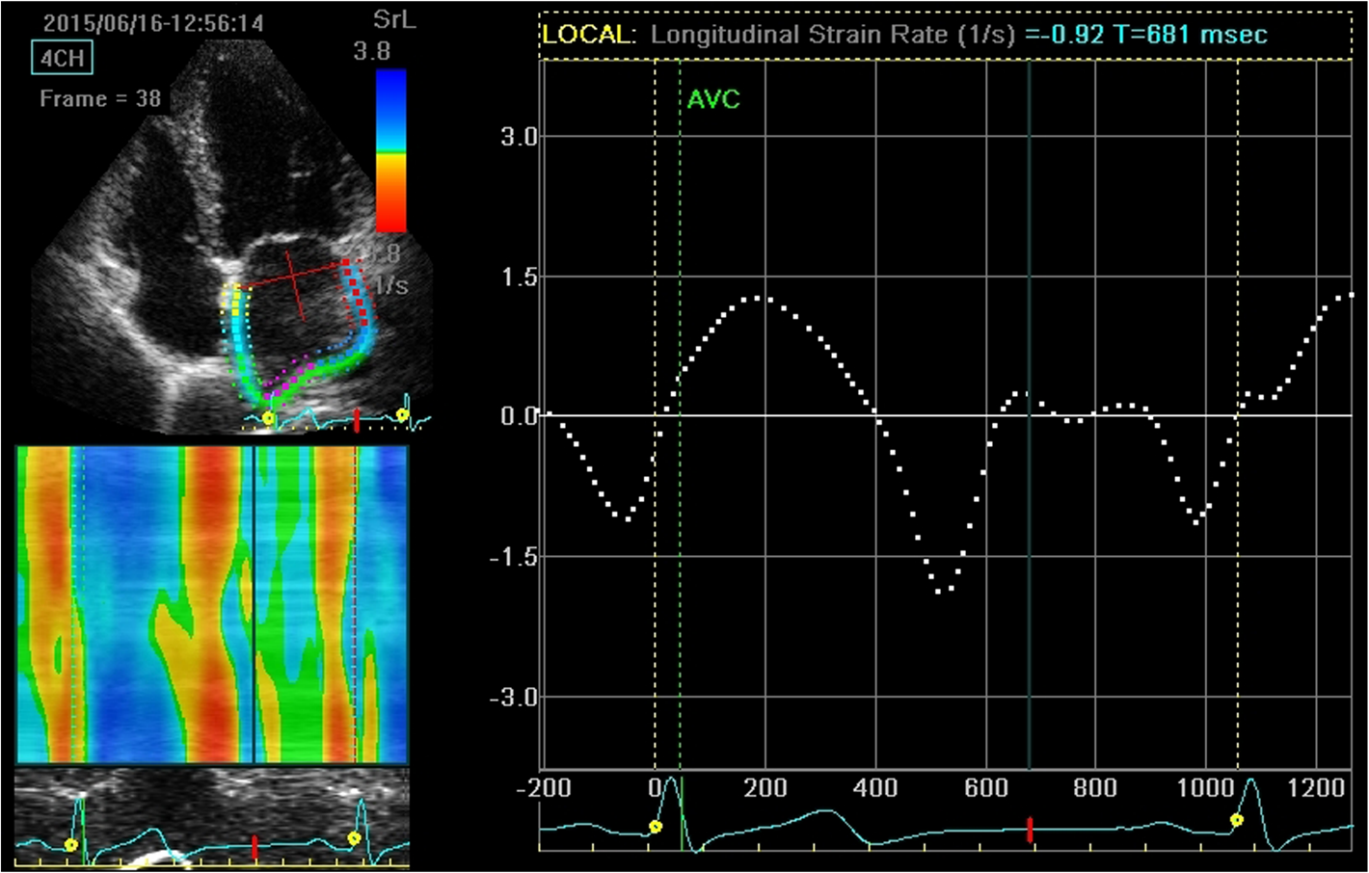
Measurement of global longitudinal left atrial strain rate from an apical four-chamber view of group A

**Fig. 2:**
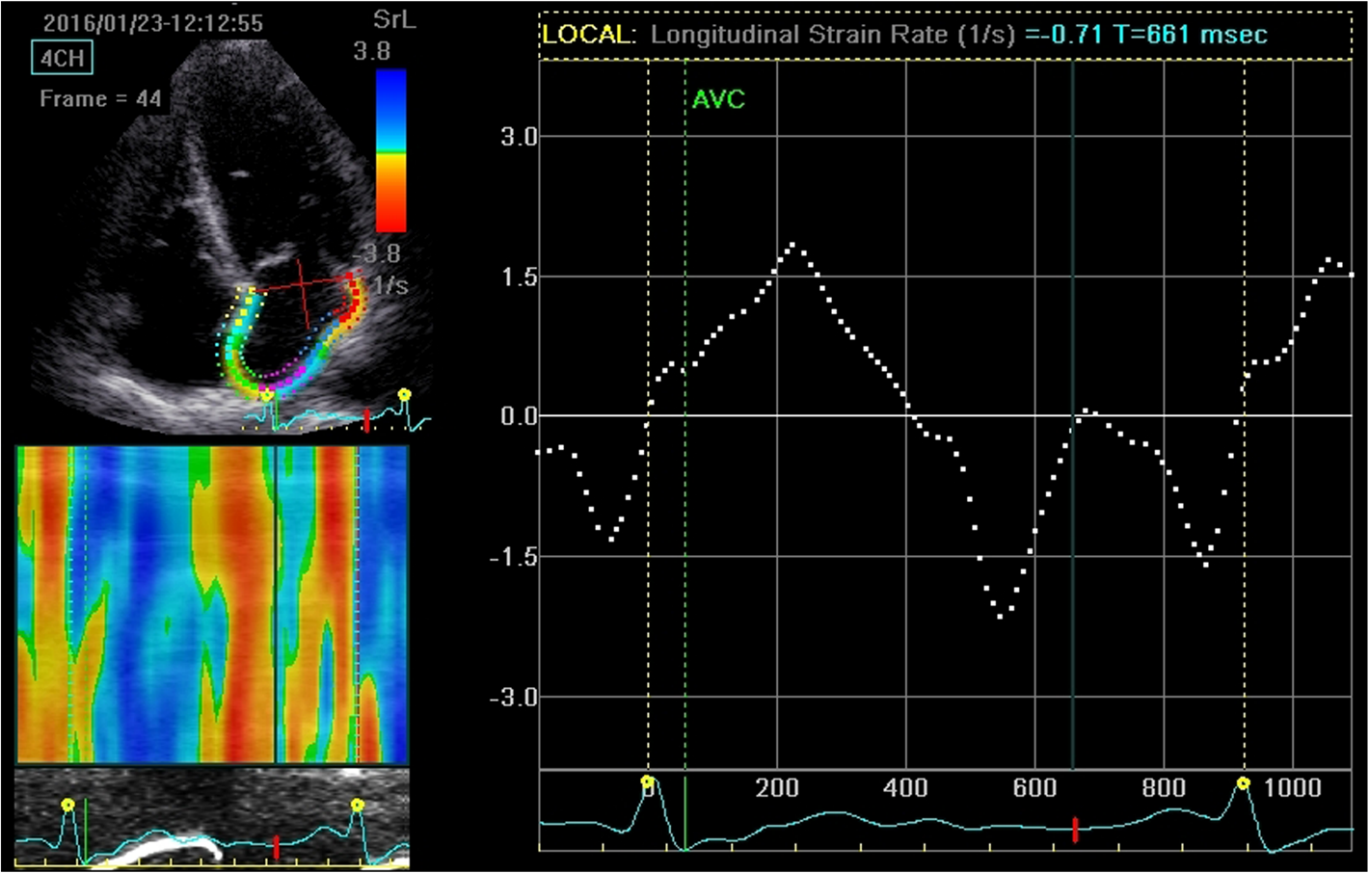
Measurement of global longitudinal left atrial strain rate from an apical four-chamber view of group B

**Fig. 3:**
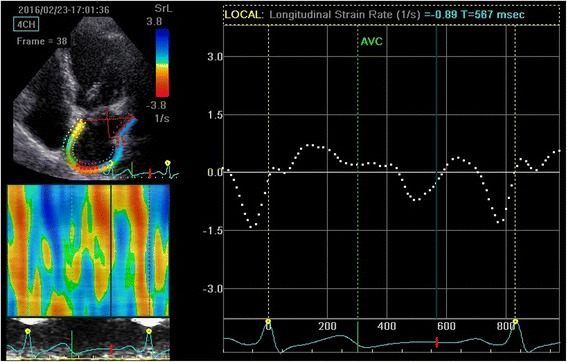
Measurement of global longitudinal left atrial strain rate from an apical four-chamber view of group C

The group B was not significant compared with the control group (all *P* > 0.05). The GLSRa of group C were significantly higher than that of group A and B (*P* < 0.01). The GLSRe, GLSs, GLSRs of group C were significantly lower than those of group A and B (all *P* < 0.01) (Seen in Table 3). The 2D-STE strain rate curves of the three groups were shown in Fig. 1, 2 and 3.

**Table 3:** Left atrial strain and strain rate

Variables	Group A	Group B	Group C
	(*n* = 30)	(*n* = 34)	(*n* = 31)
GLSs(%)	37.23 ± 5.26	35.33 ± 4.58	29.12 ± 4.83*#
GLSRs(s-1)	1.78 ± 0.26	1.71 ± 0.32	1.49 ± 0.11*#
GLSRe(s-1)	−2.12 ± 0.46	−2.05 ± 0.38	−1.57 ± 0.07*#
GLSRa(s-1)	−1.45 ± 0.11	−1.45 ± 0.24	−1.70 ± 0.16*#

*GLSs/GLSRs* the globle peak longitudinal strain rate of systolic left ventricular, *GLSRa* the globle peak longitudinal strain rate of late diastolic left ventricular, *GLSRe* the globle peak longitudinal strain rate of early diastolic left ventricular, *P <0.01 versus the control group. #*P* <0.01 versus the mild group

### Left ventricular longitudinal strain

Meanwhile, the LV longitudinal strain values was manifested in the study. The strain value of the moderate group was reduced, compared with both control group and the mild group (all P < 0.01) (Seen in Table 4).

**Table 4:** Left ventricular peak longitudinal strain

Variables	Group A	Group B	Group C
	(*n* = 30)	(*n* = 34)	(*n* = 31)
Basal(%)	−20.4 ± 4.5	−20.8 ± 4.8	−16.2 ± 4.8a#
Middle(%)	−21.8 ± 4.2	−22.3 ± 5.1	−17.1 ± 5.2*#
Apical(%)	−25.4 ± 5.3	−25.8 ± 4.7	−20.4 ± 4.6*#

*P <0.01 versus the control group. #*P* <0.01 versus the mild group

The inter- and intra-observer results revealed good reproducibility and small variability by using 2D-STE in evaluation of patients with IDA (Seen in Table 5).

**Table 5:** Inter and intra-observer analyses for LA strain and strain rate

	Intra-observer				Inter-observer			
	R	bias(%)	LOA(%)	ICC	R	bias(%)	LOA(%)	ICC
GLSs	0.80	2.39	−5.48 ~ 6.75	0.853	0.82	2.29	−6.18 ~ 4.97	0.875
GLSRs	0.85	1.28	−4.42 ~ 2.95	0.901	0.84	1.19	−5.54 ~ 4.69	0.885
GLSRe	0.81	2.14	−6.58 ~ 5.10	0.867	0.79	2.75	−8.42 ~ 3.13	0.821
GLSRa	0.90	0.65	−1.04 ~ 2.35	0.969	0.96	0.59	−0.13 ~ 1.87	0.993

*R* coefficient of determination, *LOA* limit of agreement, *ICC* intra-class correlation coefficient

## Discussion

Anemia has been shown to be an important factor in increasing cardiac output to maintain adequate oxygen supply to the tissues [6]. The transition from a high-output (compensated) state to a state of LV dysfunction (decompensated) begin at the hemoglobin below 7 g/dL in iron-deficient patients. The reduction of hemoglobin level is related to future increased morbidity and mortality [18]. So early diagnosis and treatment in iron deficiency can greatly improve quality of life and can promptly reduce hospitalization rate, unemployment rate and ultimately, reduce medical consumption [19]. Therefore it has a significant prognosis to allow for early and correct diagnosis.

The LA function is an important factor influencing cardiac output [20] by regulating the filling pressure of the left ventricular with its reservoir, conduit and pump functions. Although LA structural and functional remodeling is a barometer of LV diastolic dysfunction [21], there were no studies to reveal the changes of LA function in patients with IDA. Previous reports have shown that some disease (hypertension, atrial fibrillation, coronary artery disease, etc.) may lead to left atrial phasic dysfunction evaluated by 2D-STE [22, 23]. Additionally, the longitudinal strain and strain rate, which are inversely related to LA wall fibrosis, have been reported to be a feasible and reproducible method to assess LA myocardial function [24, 25]. So we try to evaluate the LA function in patients with IDA by 2D-STE.

In our study, there were no significant differences between group B and group A (all *P* > 0.05). None of LVDd, IVSTd and PWTd were significant among the three groups. The longitudinal strain of LV from basal to apical were decreased in the moderate group compared to the control and mild group (all *P* < 0.01). The LAD, LAVp, LAVmax, LAVmin, A and LAAEF of group C were significantly higher than those of groups A and B (all *P* < 0.01). E, E/A and LAPEF of group C were significantly lower than those of group A and B (all *P* < 0.01). We found that the conventional echocardiography parameters and strain and strain rate of LA, and longitudinal strain of LV were changed with the decrease in hemoglobin concentration. When Hb > 90 g/L, there were no obvious differences between the parameters of group B and group A compared with the control group. This suggested that the structure and function did not have obvious change in the mild group. When Hb 60–90 g/L, LAAEF and GLSRa of group C were higher than those of groups A and B. LAPEF, GLSs, GLSRe and GLSRs of group C were lower than group A and B. This meant that the LA longitudinal myocardial deformation had impaired in this stage.

However, LA abnormalities are associated with abnormal diastolic function of the LV [26–28]. LA conduit function is correlated with LV early diastolic function [29], LA reservoir function is correlated with LV systolic function [30, 31], and LA pump function is associated with LV late diastolic function [32, 33]. The result of our study had agreed to these above. At some degree, decreasing strain values means rising LV filling pressure. Based on the results, we found the following: the value of GLSs and GLSRs, the reservoir function which receives blood from the pulmonary veins during ventricular systole was decreased in the moderate group. GLSRe and LAPEF, the conduit function for transporting blood from the pulmonary veins to the LV were also decreased in the moderate group. They indicated that the movements of myocardial tissue had slowed down. These results were consistent with the longitudinal strain of LV and had indicated the LV diastolic dysfunction in the moderate group, which had associated with chronic myocardial ischemia and hypoxia [6]. Both ischemia and hypoxia may lead to LA remodeling. On the other hand, the increase of LV filling pressure could result in increasing of LA afterload, which then could affect transporting blood from the pulmonary veins to the LA. GLSRa and LAAEF, the parameters for the functional evaluation of the pump phase that established the final LV end-diastolic volume were increased in the moderate group. Perhaps it was due to the strong contraction of left atrial, which was caused by increasing length of left atrial myocardial fibers and rising LA pressure according to the Frank-Starling mechanism [34]. In addition, compared to the control and mild groups, the LV global longitudinal strain decreased in the moderate group (Table 4). Studies have manifested that longitudinal strain could be sensitive to subtle LV dysfunction, which allowed investigation of earlier stages of myocardium. In the study, the investigation of strain values in the moderate group was decreased and it was a good illustration of the sensitivity of 2D-STE in detecting earlier cardiac dysfunction. Therefore LA function and LV function are interactional. Nowadays, many clinicians cognize the importance to assess the role of left atrial function in prognosis of multiple adverse cardiovascular events, including death. After the study, most of patients in the study were made aware of the important effects of anemia to their hearts. They had all actively accepted the clinical treatment, and the result may have effectively prevented further cardiac dysfunction.

### Clinical implications

2D-STE is a sensitive tool for evaluating LA function in patients with IDA. The application of 2D-STE in patients with IDA may help clinicians to identify earlier changes of LA function. It greatly helps in early detection of abnormal LA function, even indicates clinical therapy. An aggressive therapeutic and preventive approach could improve the outcome of this disease.

### Limitations

Our study had several limitations. First, we only analyzed a part of LA wall in apical four- and two-chamber views, but in some studies, another three segments from apical three-chamber view were also included [35]. Secondly, lack of standardization could make our result incomparable with others. Thirdly, the obesity, such as lung weight may impede image quality and the unclear endocardium may also affect the result. Lastly, only 65 patients were selected in this study, the objects in the research were relatively small and only limited number of patients in extremely severe group could be obtained. In these situations, we would select more samples to study in the future.

## Conclusions

2D-STE could significantly evaluate the left atrial function in patients with IDA. And in our study, GLSs, GLSRs, GLSRe and GLSRa, the new LA function parameters, which are measured by 2D-STE, exert better potential for the accurate assessment of LA dysfunction in patients with IDA.

## Abbreviations

2D-STE: Two-dimensional speckle tracking echocardiography
E/A: Ratio of peak early and late diastolic velocities
GLSRa: The global peak longitudinal strain rate of late diastolic left ventricular
GLSRe: The global peak longitudinal strain rate of early diastolic left ventricular
GLSRs: The global peak longitudinal strain rate of systolic left ventricular
GLSs: The global peak longitudinal strain of systolic left ventricular
IDA: Iron-deficiency anemia
IVSTd: End-diastolic thickness of ventricular septum
LA: Left atrial
LAAEF: Left atrial active emptying fraction
LAD: Left atrial dimension
LAPEF: Left atrial passive emptying fraction
LAVmax: Left atrial maximum volume
LAVmin: LA minimum volume
LAVp: LA pre-atrial volume
LVDd: Left ventricular end-diastolic diameter
LVDs: Left ventricular systolic dimension
PWTd: End-diastolic thickness of LV posterior wall
